# Outcomes of surgical treatment with patterns of bacterial culture and antimicrobial susceptibility testing in cases of cervical abscessation in dogs: 82 cases (2018–2021)

**DOI:** 10.1186/s13104-023-06332-z

**Published:** 2023-05-11

**Authors:** Alexandra Walker, Nicole Amato, Jennifer Brisson, Samuel Stewart

**Affiliations:** 1 Massachusetts Veterinary Referral Hospital, 20 Cabot Road, Woburn, MA 01801 USA; 2 Ethos Discovery, 10455 Sorrento Valley Road, Suite 101, San Diego, CA 92121 USA

**Keywords:** Abscess, Canine, Cervical abscess, Cervical infection, Oropharyngeal trauma

## Abstract

**Objective:** The objective of this study was to summarize the presentation, diagnosis, and outcome for dogs surgically treated for chronic cervical abscessation following suspected or reported cervical or oropharyngeal trauma, as well as to report on culture results and antimicrobial susceptibility patterns.

**Results:** Eighty-two dogs were identified by retrospective review. Successful surgical outcome was achieved in 92.7% of dogs. Abscess recurrence was confirmed or suspected in 6/82 (7.3%) cases, and surgical intervention for abscess recurrence was performed in 4/82 (4.9%) cases. Foreign material was identified at surgery in 5/82 (6%) cases. Incisional healing complications were noted in 9/82 (10.9%) cases and required additional surgery in 5/82 (6%) cases. Twenty-three (28%) dogs had negative culture results. The results of antimicrobial sensitivity testing led to a change in antimicrobial treatment in only 9% of cases.

Surgically treated cervical abscessation carries a good prognosis with a low incidence of recurrence in this cohort (in contrast to previous reports), despite low frequency of foreign body removal or identification of the underlying cause of the abscess. Excision of chronic inflammatory tissue may not be necessary for a successful outcome, contrary to previous recommendations. Multi-pathogen infections and anaerobic infections are commonly encountered.

**Supplementary Information:**

The online version contains supplementary material available at 10.1186/s13104-023-06332-z.

## Introduction

Cervical abscessation is a commonly encountered condition in dogs with relatively little published information available in the veterinary literature regarding treatment and patient outcomes. This disease can be expensive to treat, especially in cases of abscess recurrence which is frustrating for both clients and clinicians.

Cervical abscessation in dogs is a possible sequela to penetrating oropharyngeal trauma, migrating foreign material, or external cervical wounds [1, 2]. Stick chewing and stick retrieval are well-documented causes of penetrating oropharyngeal injuries [1–3]. Acute oropharyngeal trauma secondary to penetrating stick injuries typically presents with signs of dysphagia and oral pain, whereas chronic cases typically present with ventral or lateral cervical swelling with or without a discharging sinus [1–3]. Additional clinical signs may include fever, lethargy, anorexia, coughing, gagging, neck or jaw pain, stertor, salivation, and halitosis [1, 2, 4]. Differential diagnoses for dogs presenting with signs of cervical swelling may include cervical neoplasia, cervical lymphadenitis, sialadenitis, or salivary mucocele.

Radiography, fistulography, ultrasound, computed tomography, and magnetic resonance imaging have been used clinically for the diagnosis of both acute oropharyngeal injuries and chronic cases of cervical abscessation [4–10].

Previous recommendations for the treatment of cervical abscessation in dogs, includes the meticulous identification and surgical exploration of all sinus tracts, as well as aggressive debridement of all chronic inflammatory tissue [2]. The outcome after surgical treatment of chronic cervical abscessation following oropharyngeal trauma has been reported to be guarded. One study reported an abscess recurrence rate of 18.8% following surgery with either Penrose drain placement or passive open wound drainage with no drain placement [1]. All of the dogs that experienced recurrence in this study did not have foreign material identified and removed during surgery [1]. Griffiths et al. reported an abscess recurrence rate of 30.7% (8 of 26 surviving dogs with chronic abscessation) following surgical debridement and Penrose drain placement [2]. In this group of dogs with chronic abscessation, a high proportion (61.5%) had foreign material identified and removed during surgery, but no association was found between dogs that did not have foreign material identified at surgery and the risk of abscess recurrence [2]. Nicholson et al. reported a recurrence rate of 16.7% following surgery to remove wooden fragments, in cases of chronic oropharyngeal stick injuries [4]. A recent study of 19 dogs treated surgically for deep cervical infections reported recurrence of signs within one week of surgery in 2 dogs that necessitated a change in antimicrobial therapy, and eventual resolution of clinical signs following surgery and appropriate antimicrobial therapy in 18 dogs with long term follow-up available [10].

Despite the common occurrence of this condition, previous publications have failed to document this, with only small populations of patients reflected in the veterinary literature to date. The primary purpose of this study was to report on the clinical outcome following surgical treatment of cervical abscessation in a large cohort of dogs and to report on microbial populations and antimicrobial resistance patterns.

## Materials and methods

All patients undergoing surgery at Massachusetts Veterinary Referral Hospital (Woburn, Massachusetts, USA) are recorded in a searchable, electronic document. This surgical log includes patient signalment information, diagnosis, and surgical procedure performed. The surgical log of patients that underwent surgery between November 2018 and November 2021 was searched using the following keywords: cervical, neck, swelling, and abscess. Patients that underwent surgery for a cervical abscess were included. Patients that underwent surgery for a condition other than a cervical abscess were excluded.

Patient signalment, history, physical examination findings, imaging findings, bacterial culture results, postoperative antimicrobial use, date of surgery, surgical findings, surgical technique, and outcome were recorded. The designation of acute or chronic cervical abscessation was based on criteria previously published by Griffiths et al. [2]. Cervical abscesses were defined as acute for patients displaying clinical signs for less than 7 days, and chronic for patients displaying clinical signs for 7 days or more [2].

Bacterial culture results were reviewed and recorded. The designation of multi-drug resistance was based on previously published human guidelines that have also been used in veterinary literature [11, 12]. Isolates were considered multi-drug resistant if they exhibited resistance to one or more drugs in three or more antimicrobial drug classes [11]. Isolates that exhibited intermediate susceptibility were classified as resistant to that drug.

Abscess recurrence was recorded as suspected recurrence (based on the presence of recurrent swelling or purulent discharge from the previous surgery site) or confirmed recurrence (based on surgical re-exploration or a positive bacterial culture). All clients were contacted for follow-up regarding long-term outcome and disease recurrence.

## Results

Eighty-two dogs met the inclusion criteria. Four dogs were intact females, 7 were intact males, 31 were spayed females, and 40 were castrated males. Median body weight was 24.8 kg (range 4.9 to 60.2 kg) [Fig. S1]. The median age at presentation was 5.9 years (range 0.4 to 12.9 years) [Fig. S2]. The most commonly affected dog breeds were mixed breeds (34/82), Labrador Retrievers (12/82), German Shepherds (5/82), and Golden Retrievers (5/82) [Fig. S3].

**Fig. 1:**
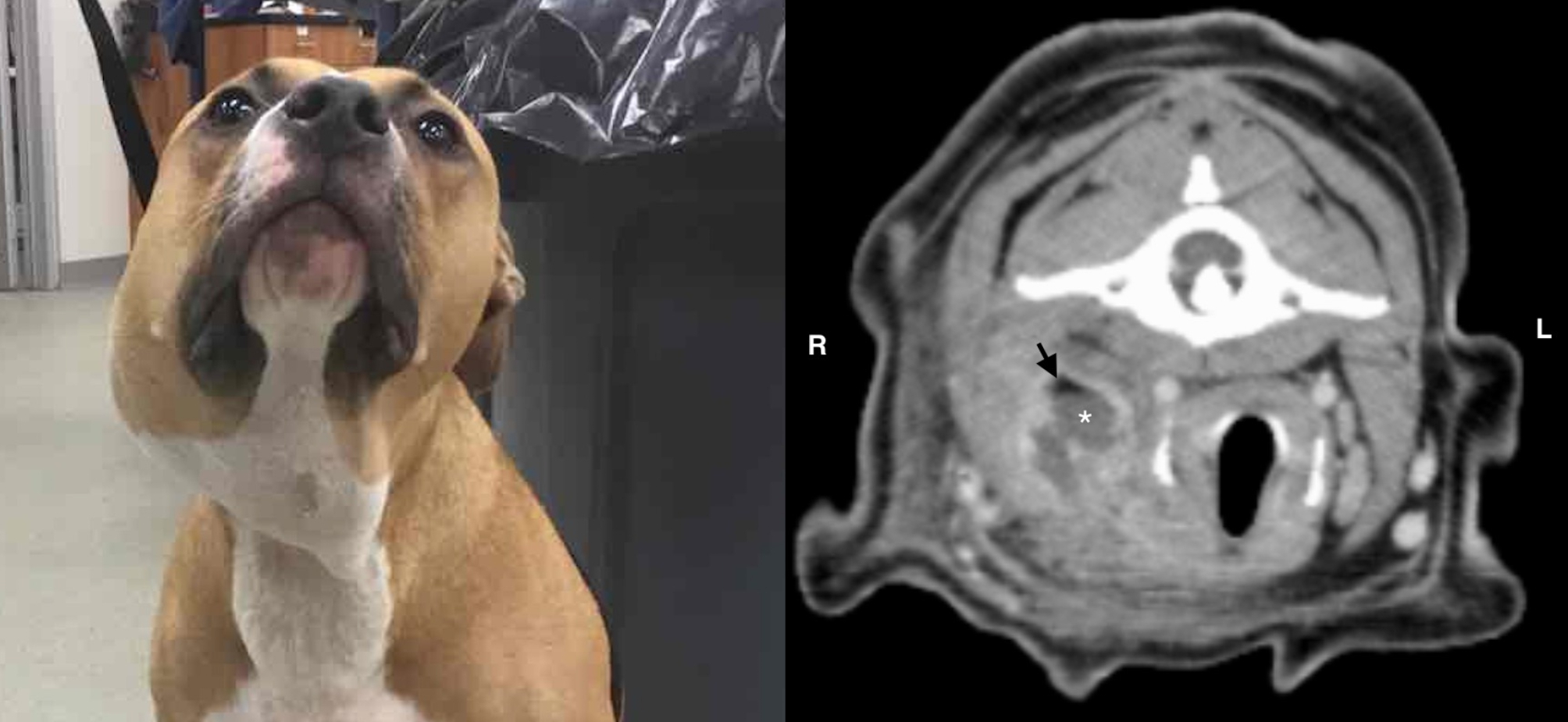
Left image: a dog showing the typical cervical swelling associated with a cervical abscess Right image: A transverse CT image at the level of the C1 vertebra showing typical findings of a cervical abscess including non-contrast enhancing fluid-attenuating pocket (*) surrounded by a contrast-enhancing periphery. Gas is present within the abscess (black arrowhead). The abscess is causing deviation of the trachea to the left side of the neck

Eighty dogs (97.5%) were presented for evaluation of cervical swelling [Fig. 1]. Additional clinical signs included fever (59.8%), inappetence (39%), lethargy (35.3%), upper respiratory signs (including coughing, wheezing, stridor and stertor − 24.4%), ptyalism (14.6%), difficulty swallowing or chewing (12.2%), labored breathing or respiratory distress (6%), and a discharging tract overlying the cervical swelling (3.6%). Nine dogs (10.9%) had been previously treated medically for cervical swelling. In these nine cases, the cervical swelling temporarily resolved with antimicrobial treatment, but recurred when antimicrobial therapy was discontinued. Twenty-seven dogs (32.9%) had a history of stick chewing and three of those dogs had a known history of oropharyngeal stick injury. Two dogs had a history of cervical bite wounds. No potential underlying cause for the cervical abscess was noted in the medical record for the remaining 53 dogs. Forty-six dogs (56.1%) were classified as acute cases, and 36 dogs (43.9%) were classified as chronic cases.

Diagnostic imaging performed prior to surgery included radiographs (2/82 cases), ultrasound (32/82), CT scan (68/82), and MRI (1/82) [Fig. 1]. Twenty-five dogs underwent both ultrasound and CT scan. Fine needle aspiration and cytology were performed prior to surgery in 39 cases. In three cases, no diagnostic imaging was performed and a diagnosis of cervical abscessation was made based on the results of the fine needle aspiration and cytology.

En bloc excision of all chronic inflammatory tissue was performed in two cases. The chronic inflammatory tissues surrounding the abscesses were not resected in the remaining 80 cases. These cases were treated by surgical exploration of the abscess, drainage of purulent material, and copious saline lavage. Patients underwent Jackson-Pratt drain placement (75.6%), Penrose drain placement (19.5%), or no drain placement (4.9%). All drains were removed 3–5 days postoperatively. Foreign material was identified during surgical exploration of the abscess in 5 dogs (6%). Foreign material identified included grass, grass awns, wood fragments, and hair.

Interestingly, distribution of cases appeared to be seasonal, with the vast majority of cases occurring in the latter half of the year with case numbers peaking in August and September [Fig. S4].

Aerobic and anaerobic culture and sensitivity testing was performed for 75/82 dogs. Five dogs had only aerobic culture samples submitted. Two dogs did not have a bacterial culture submitted. Twenty-three dogs (28.7%) had negative culture results. The remaining 57 dogs had positive culture results. Twenty-seven cultures (47.4%) grew a single isolate. Thirty cultures (52.6%) grew more than one isolate. Of the 80 dogs that had culture and sensitivity testing performed, 40 dogs (50%) had been exposed to antimicrobials within 7 days of surgery and bacterial culture submission. Dogs in this study that were exposed to antibiotics within 7 days of surgery were 1.85 times more likely to have a negative culture result, but this finding was not statistically significant (P value = 0.22).

Thirty dogs (52.6%) had purely aerobic infections and 17 dogs (29.8%) had purely anaerobic infections. The remaining 10 dogs (17.5%) had mixed aerobic and anaerobic infections. The most common aerobic bacteria isolated were *Streptococcus spp., Pasteurella spp., E. coli*, and *Staphylococcus spp* (Table 1). Anaerobes most commonly isolated were *Bacteroides spp.*, *Actinomyces spp.*, and *Fusobacterium spp* (Table 1).

**Table 1:** Summary of organisms cultured from cervical abscesses, drug resistant isolates, and isolate source

Identification	Number of isolates	Number of isolates resistant to first choice antimicrobial	Number of multi-drug resistant isolates	Isolate Source
Aerobic Isolates				
Streptococcus spp.	20			Normal Flora, skin, and oral cavity ^a, b^
Pasteurella spp.	12			Normal flora, canine oral cavity ^c^
E. -coli	7	1	1	Normal flora, GI tract ^d^
Staphylococcus spp.	7		2	Normal flora, skin ^e^
Niesseria spp.	3	1		Normal flora, canine oral cavity ^c^
Klebsiella spp.	2	1	1	Canine oral cavity ^f^
Citrobacter spp.	2	1		Ubiquitous environmental ^g^
Enterococcus spp.	1			Normal flora, GI tract ^d^
Kocuria kristinae	1	1		Normal flora, skin/mucus membranes; ubiquitous environmental h^, i^
Leclercia adecarboxylata	1			Environmental, water ^j^
Raoultella ornithinolytica	1	1	1	Environmental soil/water ^k^
Corynebacterium spp.	1	1		Normal flora, canine oral cavity ^c^
Anaerobic Isolates				
Bacteroides spp.	14			Normal flora, canine oral cavity ^b,c,d^
Actinomyces spp.	8	2		Normal flora, canine oral cavity ^c^
Fusobacterium spp.	7			Normal flora, canine oral cavity/GI tract ^c,d^
Peptostreptococcus spp.	4			Normal flora, canine oral cavity/GI tract ^c,d^
Prevotella spp.	3			Normal flora, canine oral cavity/GI tract ^c,d^
Clostridium spp.	1			Normal flora, canine oral cavity/GI tract ^c,d^
Filifactor spp.	1			Normal flora, canine oral cavity ^b^
Porphyromonas gingivalis	1			Normal flora, canine oral cavity ^c^
Unidentified anaerobic gram-negative bacillus spp.	1			
Unidentified anaerobic gram-positive cocci spp.	1	1		

^a^ Rodrigues et al., 2014; ^b^ Elliott et al., 2005; ^c^ Dewhirst et al., 2006; ^d^ Pilla and Suchodolski 2020; ^e^ Rodrigues et al., 2014; ^f^ Culham and Rawlings 1998; ^g^ Garleneau et al., 2003; ^h^ Kandi et al., 2016; ^i^ Nepolitani et al., 2019; ^j^ Keyes et al., 2020; ^k^ Hajjar et al., 2020.

Culture results prompted a change in antimicrobial coverage in 9/57 cases (15.7%) due to antimicrobial resistance of the cultured bacteria. The most common antimicrobial chosen for empirical treatment was amoxicillin clavulanate (71/82 cases). The second most common choice was the combination of amoxicillin clavulanate and enrofloxacin (6/82 cases). Other antimicrobial choices included cephalexin (1/82), amoxicillin clavulanate and clindamycin (1/82), amoxicillin clavulanate and orbifloxacin (1/82), cefpodoxime (1/82), and ampicillin sulbactam and enrofloxacin (1/82). 10% of cultured bacteria were resistant to the empirically chosen antimicrobial. Twelve isolates (12.1%) exhibited resistance to one or more antimicrobial drugs. Isolates most commonly exhibited resistance to drugs in the penicillin, cephalosporin, and tetracycline drug classes [Fig. S5]. Five isolates (5%) were classified as multi-drug resistant (Table 1).

Nine dogs (10.9%) had postoperative complications related to the surgical incision including: dehiscence (4/9), swelling (3/9), persistent purulent discharge (1/9), and peri-incisional dermatitis (1/9). The rate of incisional healing complications requiring additional surgery was 6% (5/82), with surgical revision of the incision being required in the dogs with incisional dehiscence and the dog with persistent purulent drainage.

Cervical abscesses were suspected or confirmed to have recurred in 6 dogs (7.3%). These cases are summarized in Table 2.

**Table 2:** Recurrent cervical abscesses

Recurrent abscess case #	Initial presentation	Culture results from initial surgical treatment	Previous antimicrobial treatment	Culture results from recurrent abscess	Antimicrobial treatment for recurrent abscess	Surgical debridement of recurrent abscess
1	acute	*Staphylococcus spp.* *Fusobacterium spp.*	Amoxicillin Clavulanate	*E. coli*	Amoxicillin clavulanate	Yes
2	chronic	Negative culture	Amoxicillin Clavulanate	Negative culture	Amoxicillin Clavulanate Enrofloxacin Metronidazole	Yes
3	acute	*Raoultella ornithinolytica* *Citrobacter freundii*	Amoxicillin Clavulanate Enrofloxacin	*Citrobacter freundii*	Cefpodoxime Enrofloxacin	Yes
4	acute	*Pasteurella spp.*	Initial treatment with amoxicillin-clavulanate, changed to enrofloxacin based on laboratory recommendations	*Bacillus spp.*	Amoxicillin clavulanate	Yes
5	acute	Negative culture	Amoxicillin clavulanate	*Methicillin-resistant staphylococcus aureus*	Chloramphenicol	No
6	chronic	*Pasteurella spp.* *Beta-hemolytic streptococcus spp.*	Amoxicillin Clavulanate	Not performed	Amoxicillin Clavulanate	No

The median time from surgery to recurrence was 47 days (range 7–176 days). These six dogs had abscess recurrence confirmed on bacterial culture or repeat surgical exploration. The remaining two dogs were treated medically for their cervical abscess recurrence with antimicrobial medications. Five out of six dogs had repeat aerobic and anaerobic cultures performed at the time of recurrence diagnosis. Four cultures were positive and one culture yielded no bacterial growth. Antimicrobial selection was based on repeat culture results or previous culture results in cases where repeat culture was not performed or repeat culture yielded no growth.

Fifty-five clients (67%) responded to follow up requests for information regarding abscess recurrence. The time since surgery ranged from 302 days to 1,387 days. No additional cases of abscess recurrence were identified.

## Discussion

Although severe complications related to cervical abscessation are uncommon, life-threatening upper respiratory compromise is possible due to oropharyngeal swelling, lymphadenopathy, or airway compression due to the space-occupying nature of the abscess. Five patients in this study population presented to the hospital with signs of respiratory distress. One patient required emergent intubation on initial presentation, and could not be extubated postoperatively due to persistent upper airway obstruction.

The dogs represented in this study demonstrated a low incidence of cervical abscess recurrence following surgical treatment. The cervical abscess recurrence rate following the treatments described in this study was 7.3%, which is much lower than what has been previously reported at 16.7–30.7% [2, 4]. The low incidence of recurrence prevented identification of statistically significant risk factors for abscess recurrence.

Previous recommendations for the treatment of cervical abscessation in dogs, includes the meticulous identification and surgical exploration of all sinus tracts, as well as aggressive debridement of all chronic inflammatory tissue [2]. In our study, 80/82 cases (97.5%) did not undergo resection of chronic inflammatory tissues and despite this, abscess recurrence was low. This finding suggests that aggressive debridement and removal of chronic inflammatory tissue is unnecessary, and a successful outcome can be achieved through abscess lancing, lavage, Jackson-Pratt or Penrose drain placement, and administration of appropriate culture-guided anti-microbials. Additionally, without the need for extensive resection, surgical and anesthetic time will decrease as will the risk of damage to important cervical structures during tissue debridement.

Previously reported aerobic organisms associated with penetrating oropharyngeal trauma and cervical abscesses include *Staphylococcus spp, Klebsiella spp, E. coli*, and *Streptococcus spp*. [1, 4]. Typical anaerobic microbial populations cultured from cervical abscesses thought to be secondary to penetrating oropharyngeal trauma have not been well-reported in the veterinary literature. *Bacteroides spp* and *Parvimonas micra* are previously reported anaerobic bacterial isolates from deep cervical infections in 3 dogs [10]. The negative culture rate in this patient population was 28.7%, which is lower than has been previously reported at 40-47.3% [4, 10]. The high rate of cultures that yielded no growth may represent a true sterile abscess, mishandling or delayed plating causing death of the bacterial sample, or possibly the presence of bacterial organisms that do not grow using routine culture media and lab conditions. The presence of fungal organisms is also possible, and submission of fungal cultures could be considered in future studies of this condition. Previous exposure to antimicrobials may have influenced the rate of negative cultures in this patient population, though our findings did not reach statistical significance and further studies would be required to investigate this possibility. The majority of cultured bacteria were normal flora of the canine skin, oral cavity, and gastrointestinal tract [13–18]. Other bacteria included ubiquitous environmental microbes, and those found primarily in soil and aquatic environments [19–23].

Multi-pathogen infections and anaerobic infections were commonly encountered. The degree of antimicrobial resistance may be underrepresented due to commercial veterinary laboratories providing antimicrobial recommendations based on historical data or expected susceptibility in lieu of performing antimicrobial susceptibility testing. In addition, some isolates were listed as “normal flora” and neither antimicrobial recommendations nor susceptibility testing results were provided. Despite these concerns, amoxicillin clavulanate was an appropriate antimicrobial choice in the majority of cases in this study and may be an appropriate first-line treatment for dogs presenting with cervical abscessation. Penicillins without a beta-lactamase inhibitor may not be an ideal first-line treatment given the high incidence of infections caused by anaerobic isolates, such as *Bacteroides spp*. that are known to produce beta-lactamases.

Although 53 dogs in this study had no possible cause identified in their recorded history, previous studies report that cervical abscessation is, in most cases, secondary to penetrating oropharyngeal trauma with or without retained foreign material [1, 2, 4]. In this study, foreign material was rarely identified at the time of surgery, and as noted in previous studies, identification and removal of foreign material was not necessary to achieve a successful outcome [1, 2, 4]. Some dogs in this study did have tracts of inflamed tissue between the abscess and oropharynx, esophagus, or oral cavity, identified on preoperative imaging and during surgical exploration. These findings support the supposition that penetrating oropharyngeal injury is likely a major cause of cervical abscessation in this patient population.

The reason for the seasonal distribution of cases demonstrated in this study is unknown. The dogs in this study were living in the northeastern United States. It is possible that this seasonal distribution of cases is a consequence of limited outdoor activity in the winter months due to cold weather, snow coverage, and vegetation growth patterns, resulting in dogs having limited access to sticks and plant material at this time of year.

There is currently no published literature evaluating non-surgical treatment of cervical abscessation. All dogs in this study population underwent surgical treatment, therefore conclusions cannot be drawn regarding the efficacy of medical management alone of cervical abscesses. Some dogs in this study, however, had a history of a cervical swelling that did not respond to initial antimicrobial therapy, or a cervical swelling that recurred after antimicrobials were discontinued. This finding raises additional questions regarding the medical and surgical management of these abscesses that could be evaluated in a future prospective study.

## Conclusions

Surgically treated cervical abscessation carries a good prognosis with a low incidence of recurrence, despite low frequency of foreign body removal or identification of the underlying cause of the abscess. Resection of chronic inflammatory tissue appears to be unnecessary for good clinical outcome, in contrast to previous reports. Amoxicillin clavulanate is an appropriate choice for empirical antimicrobial treatment but antimicrobial resistance is possible. To encourage good antimicrobial stewardship, aerobic and anaerobic culture with susceptibility testing to guide antimicrobial selection is recommended. A robust, prospective study would be needed for further evaluation of these findings, to provide recommendations for both non-surgical and surgical treatment options, and for identification of risk factors associated with abscess recurrence.

## Limitations

The retrospective design of this study is one of its major limitations. Potential sources of error include inaccuracies in medical records and errors in chart abstraction. Clinical follow-up was variable, and evaluation for abscess recurrence was based on client reports. It is possible that some cases of abscess recurrence were not identified due to lack of client response to the questionnaire. The aforementioned substitution of antimicrobial recommendations by reference laboratories in lieu of susceptibility testing is another potential source of error in this study.

## Electronic supplementary material

Below is the link to the electronic supplementary material.

Supplementary Fig. S1: A box-and-whisker plot showing weight data of the patient cohort.

Supplementary Fig. S2: A box-and-whisker plot showing age data of the patient cohort.

Supplementary Fig. S3: A table showing dog breeds represented in this study.

Supplementary Fig. S4: A bar graph showing the seasonal distribution of cases.

Supplementary Fig. S5: A bar graph showing the incidence of antimicrobial resistance.

## Data Availability

The datasets used and/or analyzed during the current study are available from the corresponding author on request.
