# Supplementary figures and images for: Outcomes of surgical treatment with patterns of bacterial culture and antimicrobial susceptibility testing in cases of cervical abscessation in dogs: 82 cases (2018–2021)
Source: BMC Res Notes. 2023 May 11;16:76. doi: 10.1186/s13104-023-06332-z (PMC10176722; doi:10.1186/s13104-023-06332-z)

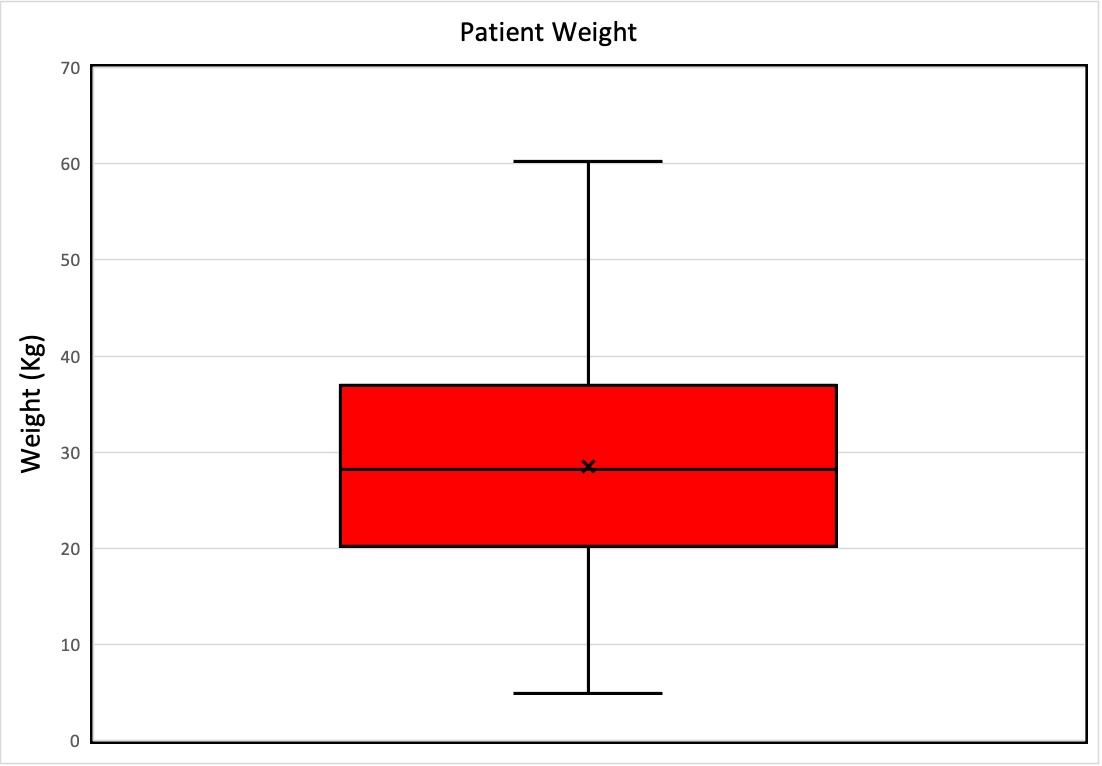

Supplement: Supplementary file 1 — Supplementary Fig. S1: A box-and-whisker plot showing weight data of the patient cohort. [file 13104_2023_6332_MOESM1_ESM.jpg]

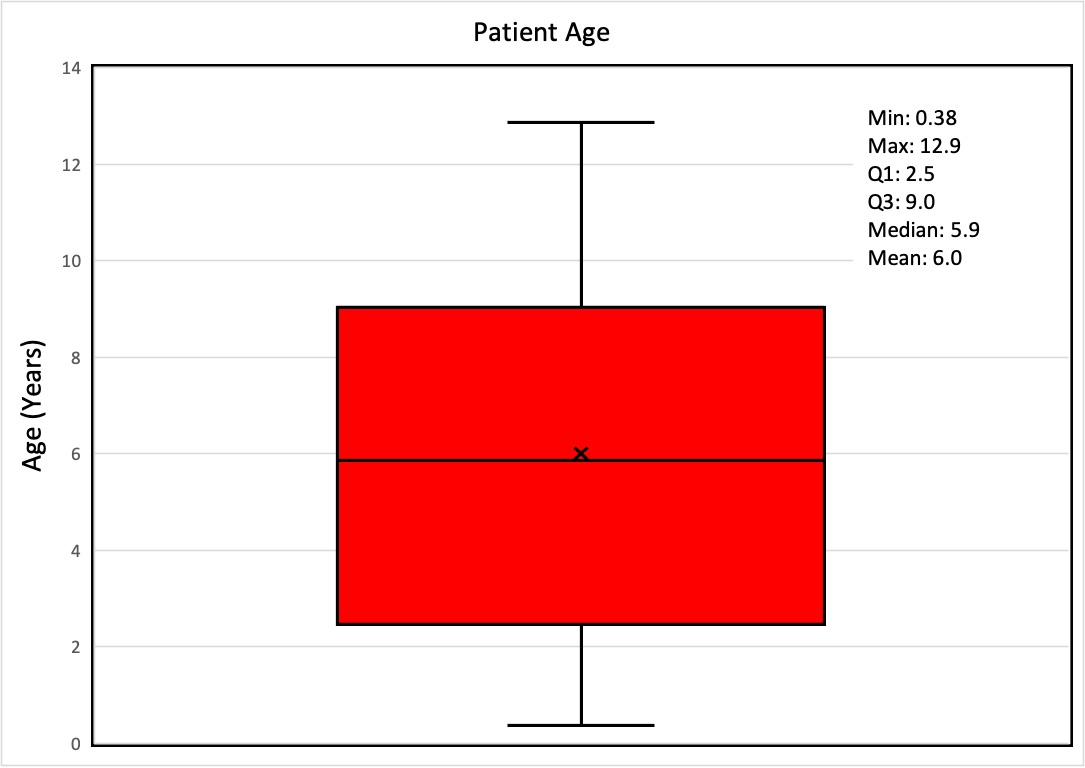

Supplement: Supplementary file 2 — Supplementary Fig. S2: A box-and-whisker plot showing age data of the patient cohort. [file 13104_2023_6332_MOESM2_ESM.jpg]

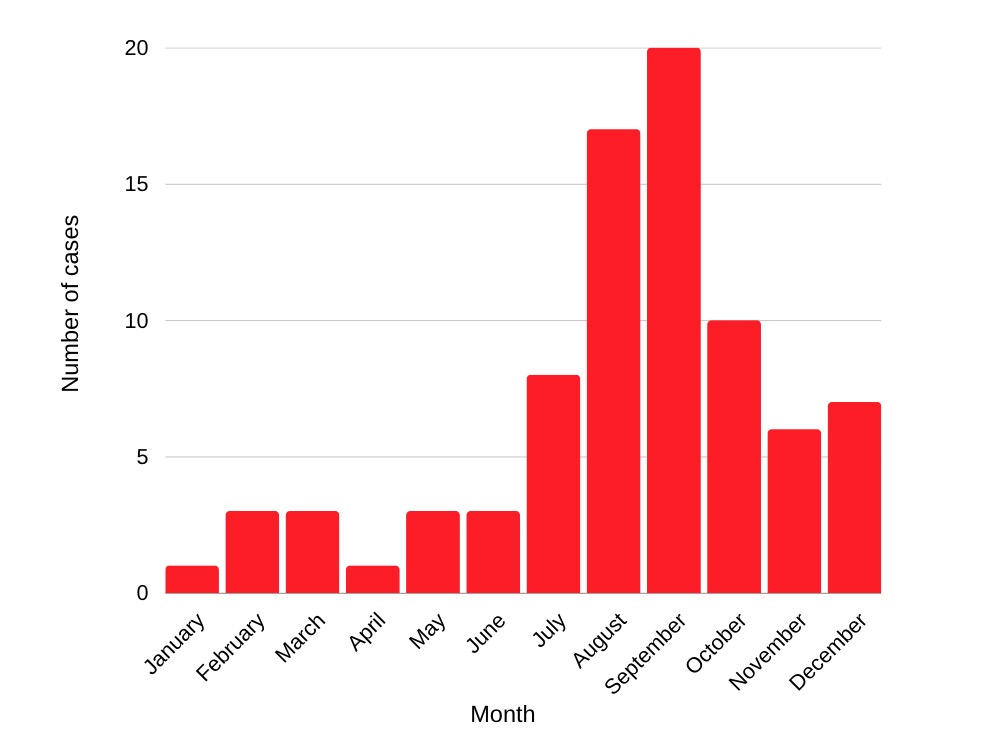

Supplement: Supplementary file 4 — Supplementary Fig. S4: A bar graph showing the seasonal distribution of cases. [file 13104_2023_6332_MOESM4_ESM.jpg]

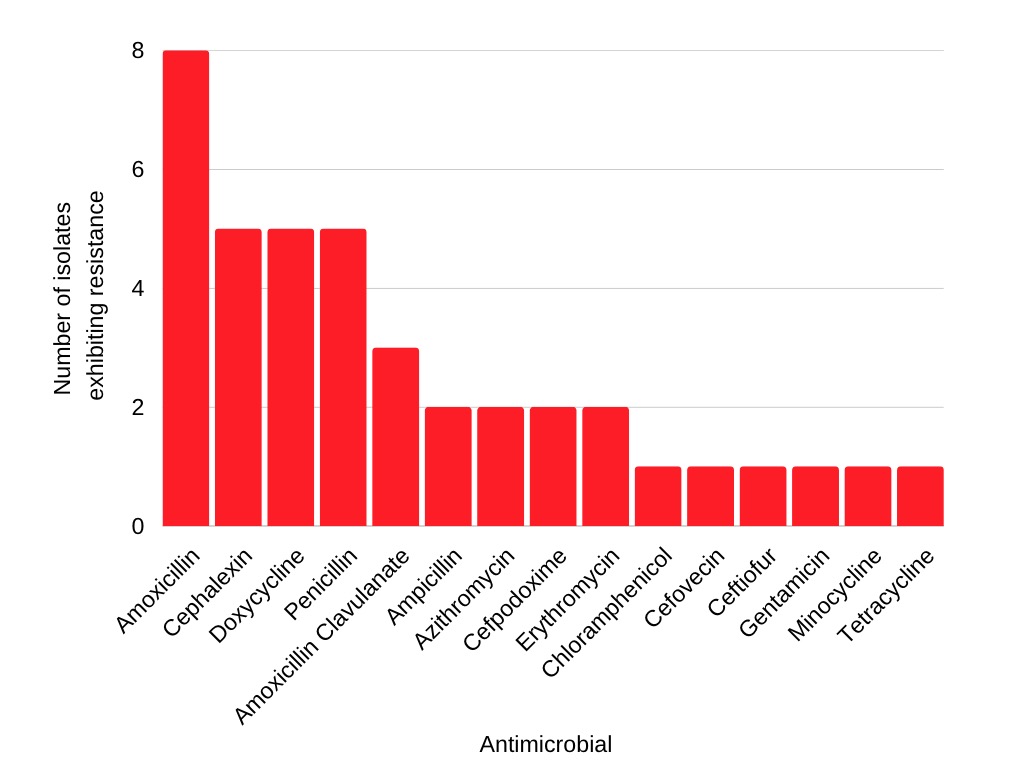

Supplement: Supplementary file 5 — Supplementary Fig. S5: A bar graph showing the incidence of antimicrobial resistance. [file 13104_2023_6332_MOESM5_ESM.jpg]
