# Supplementary material for: Outcomes of surgical treatment with patterns of bacterial culture and antimicrobial susceptibility testing in cases of cervical abscessation in dogs: 82 cases (2018–2021)
Source: BMC Res Notes. 2023 May 11;16:76. doi: 10.1186/s13104-023-06332-z (PMC10176722; doi:10.1186/s13104-023-06332-z)
Supplement: Supplementary file 3 — Supplementary Fig. S3: A table showing dog breeds represented in this study. [file 13104_2023_6332_MOESM3_ESM.docx]

| Breed | Number of cases |
| --- | --- |
| American foxhound | 1 |
| Australian shepherd | 1 |
| Beagle | 3 |
| Black mouth cur | 1 |
| Bernese mountain dog | 1 |
| Bouvier des Flandres | 1 |
| Bulldog | 1 |
| Cocker spaniel | 1 |
| Coonhound | 1 |
| Curly-coated retriever | 1 |
| Dachshund | 1 |
| French bulldog | 1 |
| Golden retriever | 5 |
| Great Dane | 1 |
| Great Pyrenees | 1 |
| German shepherd | 5 |
| German shorthaired pointer | 1 |
| Labrador | 12 |
| Mixed breed | 34 |
| Old English sheepdog | 1 |
| Poodle | 2 |
| Portuguese water dog | 1 |
| Rottweiler | 1 |
| Staffordshire bullterrier | 1 |
| Springer spaniel | 1 |
| Whippet | 1 |
| Yorkshire terrier | 1 |
